# Quality Improvement Project to Reduce Wait Time for Pediatric Gastrointestinal Procedures

**DOI:** 10.1097/pq9.0000000000000900

**Published:** 2026-07-29

**Authors:** Jennifer M. Colombo, Nicholas A. Clark, Laura E. Shroyer, Dustin J. Hahn, Jonathan Patterson, Kenneth J. Sam

**Affiliations:** From the * Division of Pediatric Gastroenterology, Department of Pediatrics, Children’s Mercy Kansas City, Kansas City, Mo.; † School of Medicine, University of Missouri-Kansas City, Kansas City, Mo.; ‡ Ambulatory Services, Children’s Mercy Kansas City, Kansas City, Mo.; § Perioperative Services, Children’s Mercy Kansas City, Kansas City, Mo.; ¶ Performance Improvement, Children’s Mercy Kansas City, Kansas City, Mo.

## Abstract

**Introduction:**

Delays in care can lead to poor health outcomes and suboptimal patient and family experiences. We aimed to reduce the average wait time for gastrointestinal endoscopic procedures (procedure request to procedure completion) from 145 to 15 days (a 90% reduction) within 4 months (November 2023 to March 2024).

**Methods:**

A3 problem-solving methodology was used. The primary intervention was a multiday, multidisciplinary improvement workshop. Outcome measures included (1) procedure request date to procedure completion date, and (2) favorable patient satisfaction score for timeliness of access to care. Process measures included (1) procedure request date to date procedure scheduled by staff, and (2) date procedure scheduled by staff to procedure completion date. The balancing measure was the number of procedures scheduled per weekday. Control charts assessed the impact of interventions.

**Results:**

Following the primary intervention (October 2023), special cause improvement was seen for all but the balancing measure. Outcome measure 1 achieved a sustained reduction in mean monthly wait time from 145 to 26.4 days (an 82% reduction), whereas outcome measure 2 achieved sustained improvement in patient satisfaction from 39.7% to 50.2% (a 26.4% relative improvement). Process measures 1 and 2 had sustained reductions from 56.5 to 14.7 days (a 74% reduction) and 89.1 to 20.7 days (a 77% reduction), respectively. The balancing measure also remained unchanged at a monthly average of 11.7 procedures scheduled per weekday.

**Conclusions:**

Using A3 problem-solving methodology, we achieved an 82% sustained reduction in endoscopic procedure wait times with a concurrent 26.4% improvement in patient satisfaction. Procedures are now routinely completed within 1 month of the request date.

## INTRODUCTION

Lack of timely access to care can contribute to prolonged patient distress, poor patient and family experiences, and adverse health outcomes.^1^ Multiple pediatric gastroenterology guidelines support the role of endoscopy (eg, esophagogastroduodenoscopy and colonoscopy) in the diagnosis of pediatric gastrointestinal (GI) diseases,^2–7^ and delays in these procedures may impair the ability to diagnose digestive disorders and initiate treatment.^8^ Factors that may contribute to longer wait times include lack of human resources, complex ordering algorithms, inefficient scheduling processes, lack of appropriate space, and communication errors. Longer wait times for endoscopic procedures may result in nonattendance at scheduled appointments, further compounding the problem.^9^ The international Pediatric Endoscopy Quality Improvement Network (PEnQuIN) has established clinically meaningful metrics to promote safety and quality in pediatric endoscopic procedures.^10^ To date, there are no accepted pediatric standards for the time from GI endoscopic procedure request to procedure completion, except in the cases of urgent or emergent procedures.^11^

As a result of operating room (OR) construction and pandemic-related staffing challenges, our patients began experiencing prolonged wait times for endoscopic procedures, with an average wait time of 145 days from the date of procedure request to procedure completion. Further, only 41% of families were satisfied with the wait time when asked if they were able to get an appointment as soon as they wanted. Our global aim was to ensure timely access to GI endoscopic procedures. Our SMART aim was to reduce the average wait time for a routine elective (nonurgent, nonemergent) GI endoscopic procedure (time from procedure request to procedure completion) from 145 to 15 days (90% reduction) within 4 months (November 2023 to March 2024). Our secondary aim was to increase the monthly percentage of patient satisfaction survey favorable responses for timeliness of access to care from 39.7% to 60% (~50% increase) within the same 4-month timeframe.

## METHODS

### Setting and Context

Our institution is a 390-bed, freestanding children’s hospital located in the Midwest. It has 22 ORs across 2 campuses (primary and satellite). The hospital performs an average of approximately 3,000 GI endoscopic procedures each year. The primary GI clinic is located at the main hospital campus but also has additional GI clinics operating at several satellite locations across the region. The GI clinics host an average of 21,000 outpatient visits annually.

During the preintervention state, GI providers placed orders for GI procedures via a request queue located in the electronic medical record (EMR; PowerChart; Oracle Corporation; Nashville, TN). GI nursing staff were responsible for scheduling the GI procedures. This process included an in-depth chart review of each patient to determine the appropriate location for the GI procedure (high risk at the primary campus versus low risk at the satellite campus). Once the chart review was complete, GI nurses would call legal guardians, or patients if 18 years of age and older, to request an OR date through the perioperative OR scheduling office. The OR scheduling office would then schedule the procedure date.

From April 2022 to October 2023, hospital construction resulted in OR closures (4 rooms). Concurrently, the global pandemic caused a shortage of anesthesia providers, nurses, and GI technologists. These barriers, along with inefficient procedure scheduling, caused a prolonged wait time for GI procedures. By June 2023, the patient waitlist for scheduling their procedure had increased to a high of 533 patients, and the average wait time from procedure request to scheduling of the procedure was 56.6 days, with an additional 89.1 days of waiting from the date the procedure was scheduled to the date the procedure was completed (nearly a 5-mo total wait time).

In July 2023, a section of the perioperative services was formed to centralize all surgery scheduling efforts across the institution. Creation of the centralized scheduling team aligned standards for OR scheduling across perioperative services. The new process included standardization for requesting OR procedures, as well as outlining anesthesia guidelines for which patients should be scheduled at which OR location based on patient acuity (primary hospital versus satellite hospital).

### Intervention Planning

Leading up to project initiation, perioperative services and the GI division discussed opportunities for improving wait times and streamlining scheduling processes. A focused improvement workshop (FIW) was planned. An FIW is a multidisciplinary, 3-day workshop (held October 2023) to brainstorm and develop countermeasures and was led by an experienced process improvement staff member (K.J.S.). Stakeholders in the FIW included more than 30 team members from GI, anesthesia, nursing, perioperative services, scheduling, medical informatics, and process improvement. The FIW used the A3 methodology to carry out the baseline investigation and implementation plan. A3 is a structured problem-solving methodology.^12^ Primary drivers identified for improvement were standardizing EMR GI procedure order requests, streamlining GI procedure scheduling processes, provider engagement, and improving training and education.

### Interventions

Eighteen countermeasures were initially identified, with countermeasure implementation determined through development of a prioritization matrix. All selected countermeasures were developed and refined during the FIW and implemented shortly thereafter (November 2023). Confirmations to ensure reliability of implementation included visualizing the process (gemba walks), abnormality trackers, and monthly review by both the primary project and oversight teams.

#### Scheduling Template Enhancement

GI and centralized surgery scheduling used 2 different tools to schedule procedures. The primary tool used by centralized surgery scheduling was the EMR messaging inbox. Meanwhile, the GI team used an EMR GI procedure order. A new way of ordering GI procedures was needed to align GI and centralized scheduling efforts. The new GI scheduling template was created by medical informatics using a human factors approach through obtaining input from all end users and beta-testing in real time during the FIW. A side-by-side time trial was conducted comparing the old versus new scheduling templates and found that a similar time was required for both (30 versus 45 s, respectively), despite minimal familiarity with the new template. Key components of the new template included procedure location based upon patient risk (primary versus satellite campus), scheduling logistics (anticipated procedure duration, priority, hospitalization postprocedure, patient diagnoses, and procedural billing codes), and patient and provider needs (preprocedural documentation completion, specialized equipment needs, anesthesia considerations such as American Society of Anesthesiologists class and body mass index). When feasible, parts of the template were prefilled with standard language so GI providers would not need to add this information (eg, standard OR time for specific GI procedure, CPT codes for each GI procedure, additional equipment needed for special procedures, standard biopsy sites for routine procedures). GI providers completed the template when requesting a procedure, with an option for secondary review by an anesthesia team member if questions arose. To ensure reliability of new template use, the old GI ordering system was deactivated. Since implementation, more than 3 iterations have been made to improve the template based on feedback and recurring themes.

#### Scheduling Process Refinement

Managing the list of patients waiting to have a GI procedure scheduled was cumbersome. Before the new template development, the list did not contain details necessary to efficiently schedule a procedure, such as procedure location based upon patient risk or acuity (high versus low), current procedural terminology code for billing, prioritization of procedure, or the name of the provider who requested the procedure. GI nurses spent hours conducting manual chart reviews to assign patients to the medically appropriate procedural location. Twelve full-time GI procedure nurses were needed to assist with GI procedures and schedule GI procedures. The new template developed during the FIW decreased end user burden and captured all the necessary scheduling information in a single place. The task of GI procedure scheduling was transitioned to the perioperative services centralized surgery scheduling team, which consisted of 3 perioperative schedulers for GI procedures. These changes, along with the new scheduling template, streamlined the scheduling process.

#### Provider Education

With substantial changes to the process, we paid close attention to educating team members before implementation. Anesthesia created standardized guidelines to aid in appropriate location selection. The new process was taught to frontline providers (October 2023). Real-time and just-in-time coaching was provided with updates as needed when process changes were made.

### Study of Interventions and Analysis

Control charts were used to display monthly data over time. Data were collected from both OR locations, with 10 charts manually reviewed each month and averaged into a single monthly data point using continuous data. Therefore, Xbar-R charts were developed in QI Macros (KnowWare Inc., Denver, CO). As patient satisfaction survey responses were assessed as favorable or unfavorable, a monthly percentage was generated, and a p-chart was used for that measure. Montgomery control chart rules^13^ were used to determine common versus special cause variation. Following FIW, monthly team meetings, including both project and oversight teams, were held for 4 months to review current performance, process updates, and suggest next steps, as necessary.

### Measures

#### Outcome Measure 1: Procedure Request to Completion

The first outcome measure was the mean number of days between the procedure request date by the GI provider and the date of procedure completion. This measure is a bundled measure of the 2 process measures (outcome measure 1 = process measure 1 + process measure 2; lead time = cycle 1 time + cycle 2 time). Ten charts were selected at random each month to calculate the monthly mean lead time, and these values were then displayed on an Xbar-R control chart.

#### Outcome Measure 2: Patient Satisfaction

The second outcome measure was the mean monthly percent of favorable responses to a patient satisfaction survey (“Yes, definitely”) for the receipt of timely access to care. Responses were to the question: “Did you get an appointment as soon as you wanted?” regarding GI procedural access. Monthly responses were aggregated into a single percentage per month and displayed on a p-chart. Our institution updated its satisfaction survey, and this question was no longer included after June 2024. Therefore, the final data point for this measure is June 2024.

#### Process Measure 1: Procedure Request to Scheduled

The first process measure was the mean monthly time in days from when a procedure was requested (ordered) by a GI provider to when it was officially scheduled by a staff member (cycle time 1). This measure constituted the first cycle in our process’ lead time. This measure was chosen to determine how quickly patients were being contacted to schedule their procedure. This process measure highlights the schedulers’ timeliness in contacting families to schedule their procedure to improve outcome measure 1. Data were extracted from the EMR and displayed monthly on an Xbar-R control chart, as data were obtained from manual chart review of 10 random encounters per month.

#### Process Measure 2: Procedure Scheduled to Completion

The second process measured the monthly time in days from when a procedure was scheduled by a staff member to when it was completed in the OR. This measure constituted the second and final cycle time in our process’ lead time. This measure was chosen to determine when patients were added to the procedure schedule based on availability. This process measure emphasizes whether schedule availability should be adjusted to improve outcome measure 1. Data were extracted from the EMR and displayed monthly on an Xbar-R control chart, as data were obtained from a manual chart review of 10 random encounters per month.

#### Balancing Measure: Procedures Scheduled per Day

The balancing measure was the mean monthly number of procedures scheduled by staff per weekday. This measure was chosen to monitor the burden on the scheduling team with the new streamlined process. Data were extracted from the EMR and were displayed monthly on an XmR control chart.

This study was deemed quality improvement (QI) by our Office of Research Integrity and exempt from formal review.

## RE0SULTS

### Outcome Measure 1: Procedure Request to Completion (Lead Time)

**Fig. 1.**
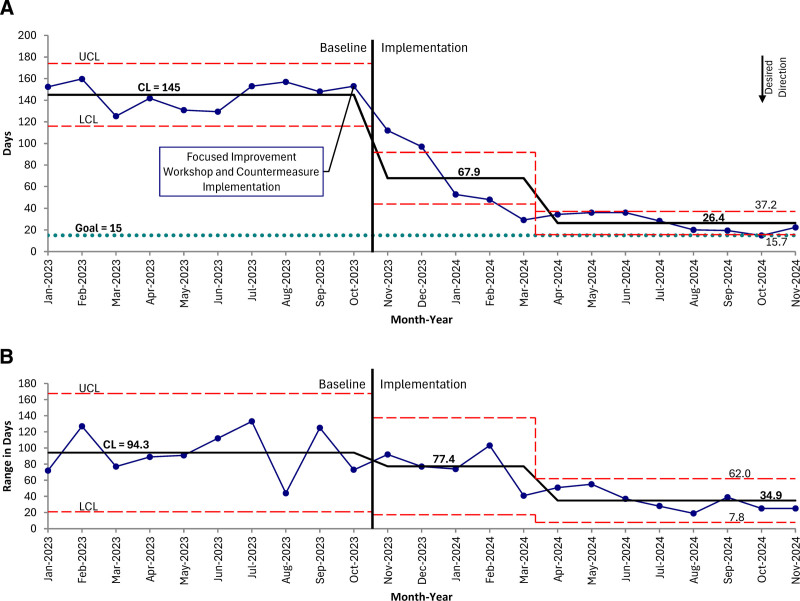
Outcome measure 1. Xbar-R paired charts displaying lead time in days from procedure request to procedure completion. Ten patients per subgroup (data point). A, X chart displaying mean monthly lead time. B, R chart displaying variation between X-chart data points. Less variation between data points suggests a more “in control” system (ie, March 2024 to November 2024). CL, centerline; LCL, lower control limit; UCL, upper control limit.

A total of 230 charts were manually reviewed (90 baseline; 140 implementation). Special cause improvement occurred starting in November 2023, immediately following the FIW, with a downward trend of 6 data points (October 2023 peak to March 2023 trough) and with the November 2023 data point, and all subsequent data points, being less than 3σ (sigma)/below the lower control limit of the baseline period (145–67.9 d). Special cause was again observed in April 2024, with 8 points below the preceding centerline and with April 2024, and all subsequent data points, being less than 3σ (sigma)/below the lower control limit of the preceding period (67.9–26.4 d; April 2024–November 2024; Fig. 1). This resulted in a total of 82% relative reduction from baseline to the end of the implementation phase. Also starting in March 2024, the accompanying R chart displayed very “in-control” data (minimal variation between data points).

### Outcome Measure 2: Patient Satisfaction

**Fig. 2.**
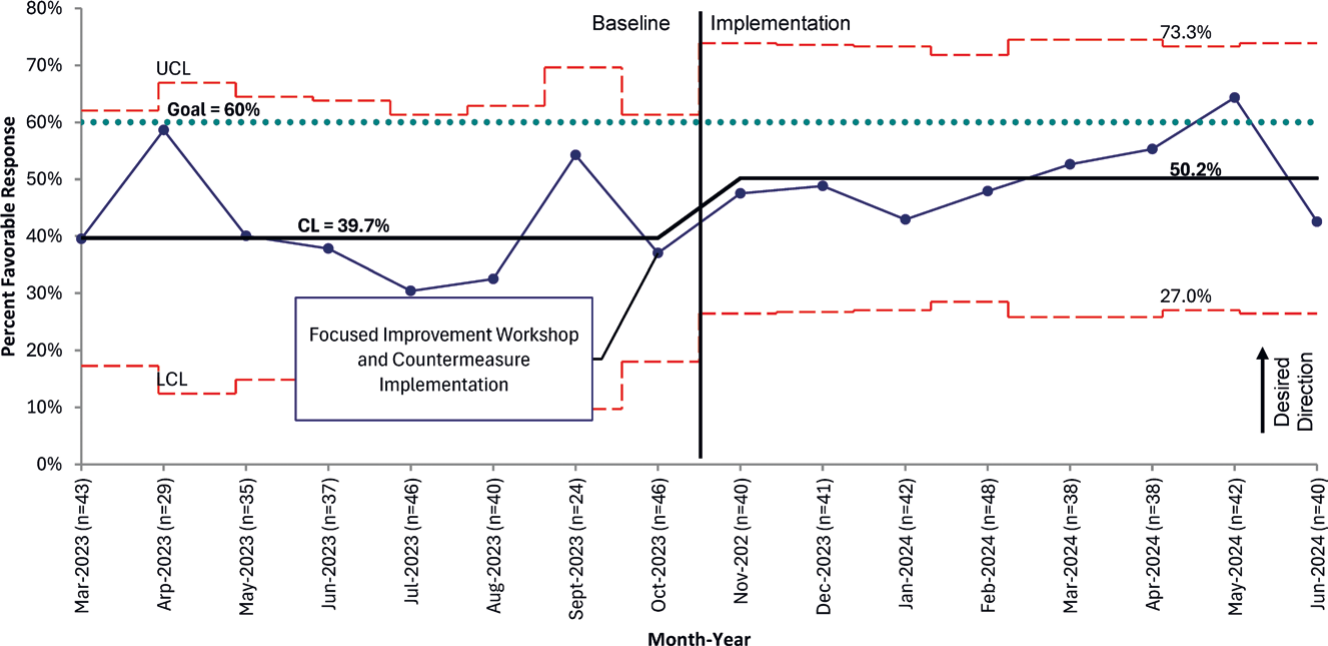
Outcome measure 2. P-chart displaying the monthly percentage of favorable responses to patient satisfaction surveys regarding receipt of timely access to care. Our institution discontinued this question on the satisfaction survey in July 2024. CL, centerline; LCL, lower control limit; UCL, upper control limit.

Special cause improvement was observed starting in November 2023, immediately following the FIW, with 8 points above the baseline centerline (39.7%– 50.2%) for a 26.4% relative increase (Fig. 2).

### Process Measure 1: Procedure Request to Scheduled

**Fig. 3.**
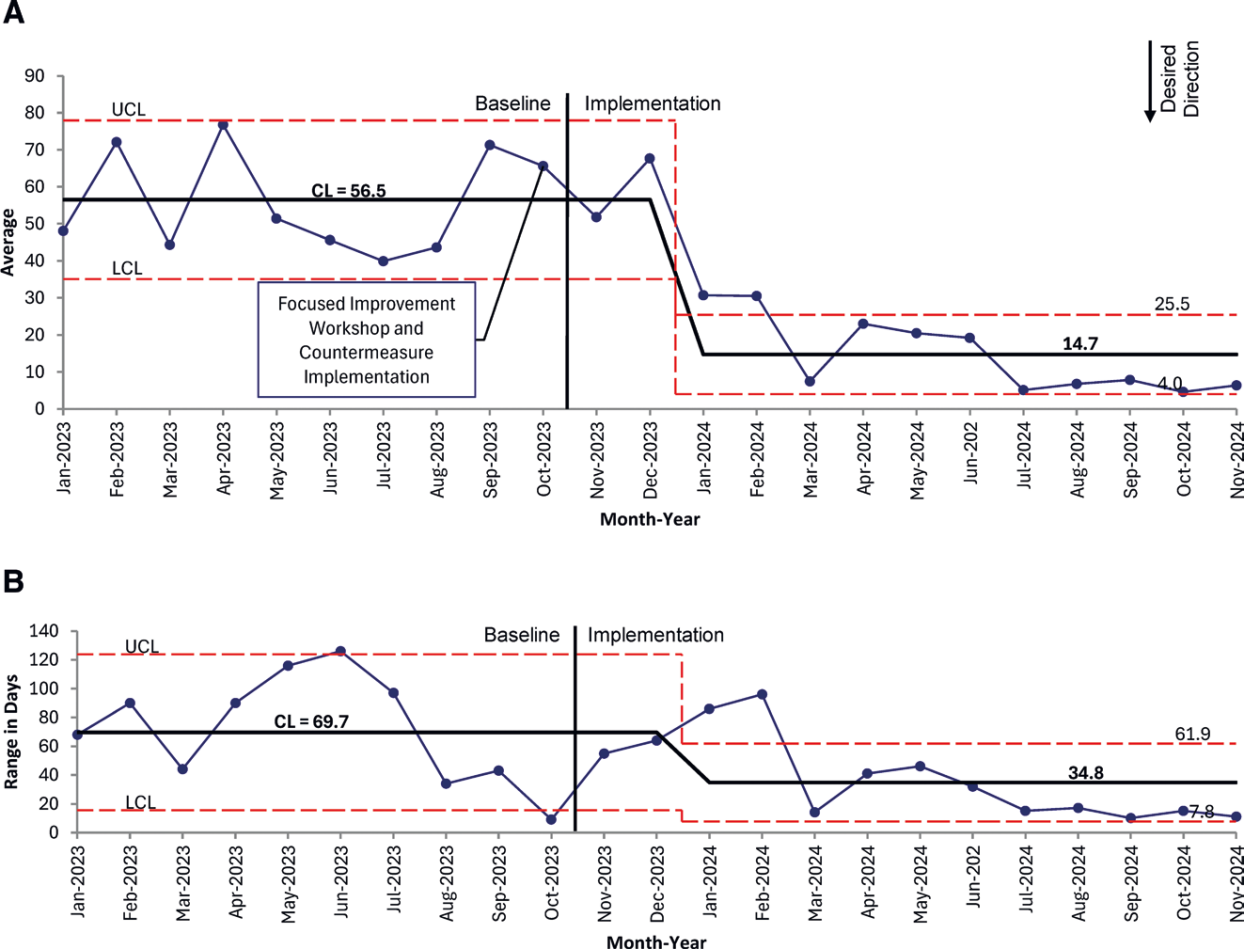
Process measure 1. Xbar-R paired charts displaying cycle time 1, defined as the mean monthly time in days from procedure request to procedure scheduled. Ten patients per subgroup (data point). A, X chart displaying mean monthly cycle time 1. B, R chart displaying variation between X-chart data points. Less variation between data points suggests a more “in-control” system (ie, March 2024 to November 2024). CL, centerline; LCL, lower control limit; UCL, upper control limit.

Special cause improvement occurred starting in January 2024, 3 months following the FIW, with all subsequent data points being less than 3σ (sigma)/below the lower control limit of the baseline period while also displaying 8 points below the baseline centerline (56.5–14.7 d) for a 74% relative reduction (Fig. 3). Improvement was sustained for the remainder of the data collection period. Also starting in March 2024, the accompanying R chart displayed more “in-control” data.

### Process Measure 2: Procedure Scheduled to Completion

**Fig. 4.**
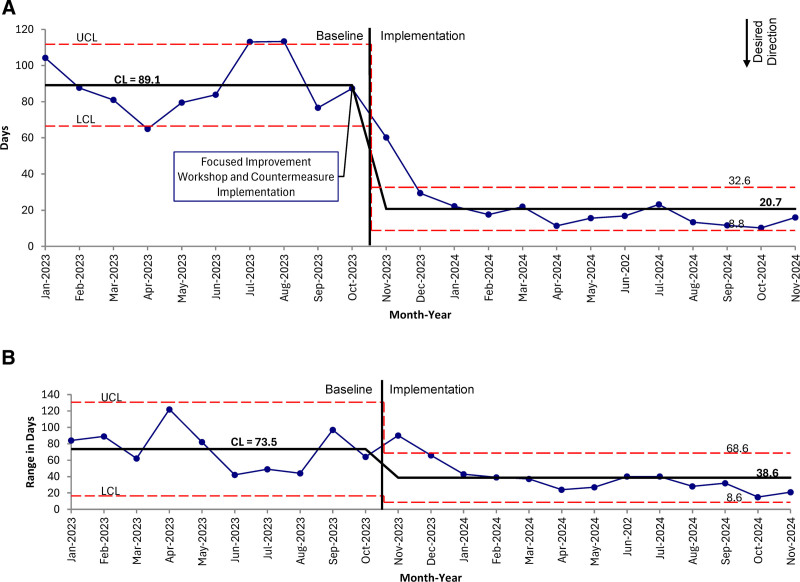
Process measure 2. Xbar-R paired charts displaying cycle time 2, defined as the mean monthly time in days from procedure scheduled to procedure completion. Ten patients per subgroup (data point). A, X chart displaying mean monthly cycle time 2. B, R chart displaying variation between X-chart data points. Less variation between data points suggests a more “in-control” system (ie, January 2024 to November 2024). CL, centerline; LCL, lower control limit; UCL, upper control limit.

Similarly, special cause improvement occurred starting in November 2023, immediately following the FIW, with all subsequent data points being less than 3σ (sigma)/below the lower control limit of the baseline period while also displaying 8 points below the baseline centerline (89.1–20.7 d) for a 77% relative reduction (Fig. 4). Improvement was sustained for the remainder of the data collection period. Also starting in January 2024, the accompanying R chart displayed more “in-control” data.

The sum of the centerline values of process measures 1 and 2 is greater than outcome measure 1’s final centerline value. This is because the centerline shifts in each of the measures did not occur at the same time. When individual data point values for process measures 1 and 2 are added together, they equal the corresponding data point value for outcome measure 1. For example, November 2024 data points: P1 (6.4 d) + P2 (15.9 d) = O1 (22.3 d).

### Balancing Measure: Procedures Scheduled per Day

Special cause variation was not appreciated, and this remained at a monthly mean of 11.7 GI procedures scheduled per weekday. This indicates that the new process did not negatively impact scheduling processes.

## DISCUSSION

Pairing A3 problem-solving methodology^12^ with an FIW, we significantly reduced the wait time for GI procedures at our institution. An 82% reduction in lead time from the procedure request to completion was achieved by focusing on improving individual cycle times by 74% and 71%, respectively. GI endoscopic procedures are now routinely completed within 1 month from the request date, and patient satisfaction scores have risen by 26%. We reduced the backlog of patients waiting for GI procedures while also managing new patient requests for procedures. Additionally, we did not observe a negative impact on our scheduling team despite the need to schedule more procedures each weekday. Kaplan et al^14^ identified contextual factors with the strongest effects on measures of QI success, including resource availability, QI team leadership, team QI skills, and microsystem motivation, QI culture, and QI capability. Although not every institution may have experts trained in leading FIWs, the concepts applied and countermeasures implemented to address our problem could be adapted to other institutions hoping to identify unnecessary waste in processes and reduce delays in patient care.

We are unaware of other QI publications specifically addressing lead time reduction in GI procedures across multiple locations. Van Veen-Berkx et al^15^ were able to improve OR scheduling and use through collaboration of cross-functional teams. We observed similar benefits by combining separate but parallel workflows (GI and surgery scheduling) into a single, streamlined, centralized scheduling workflow. Most GI procedures are diagnostic in nature, and although there are no published pediatric standards for timeliness of GI procedures, delays in these procedures and the subsequent care can result in harm to the patient.^1,8^ Taking a multidisciplinary approach to standardizing processes for efficiency can result in more timely care. This is evidenced by Politi et al,^1^ who examined root cause analyses to identify contributing factors to delays in care and found supporting evidence for standardization of care processes and procedures to reduce delays and improve care. Additionally, Naiker et al,^16^ in a systematic review, concluded that strategies which align resources, increase operational efficiency, and streamline processes can reduce outpatient waiting times. Improving outcomes in improvement projects depends on communication between team members, regular meetings to facilitate implementation, and adapting a problem-solving framework.^17^ We found this was true with our efforts as well.

Our study does have some inherent limitations. This improvement project was completed at a large, freestanding children’s hospital with an abundance of support and resources that may not be available at other institutions. Therefore, countermeasures implemented, and results experienced, may not be generalizable. Another limitation is that only 10 patients were included per data point due to the resources needed to conduct manual chart review and data collection. Despite the risks associated with a smaller “n” per data point, we were still able to achieve sustained special cause variation with minimal variation between data points. Patient satisfaction data are difficult to interpret. Although we observed an improvement in satisfaction related to timeliness in the months following implementation, we did see our final data point fall below the centerline. During project design, we were unaware that the survey question tied to outcome measure 2 would be removed from the satisfaction survey starting July 2024, and therefore, we are unable to determine if June 2024 is merely common cause variation or the beginning of a regression to the baseline mean. Although the addition of 4 OR suites coincided with our project’s implementation phase and may have made a small impact on our project’s results, we do not feel it played a significant role, as those rooms were allocated across all surgical specialties and were not reserved for GI procedures only. Finally, we did not stratify data by race, ethnicity, language, or other social drivers of health and, therefore, may have inadvertently maintained or worsened a disparity.

## CONCLUSIONS

Using A3 problem-solving methodology, we significantly reduced wait times for GI procedures at our organization while improving patient satisfaction. This was achieved by standardizing processes through multistakeholder input, human factors design, and a focused improvement workshop. Future work involves continuing to shorten the cycle and lead times, as well as spreading this work to other surgical specialties throughout our organization.

## ACKNOWLEDGMENTS

The authors thank the Focused Improvement Workshop team, Management Guidance team, and sponsors.
